# Chemical Absorption of CO_2_ Enhanced by Nanoparticles Using a Membrane Contactor: Modeling and Simulation

**DOI:** 10.3390/membranes9110150

**Published:** 2019-11-11

**Authors:** Nayef Ghasem

**Affiliations:** Department of Chemical & Petroleum Engineering, UAE University, Al-Ain 15551, UAE; nayef@uaeu.ac.ae

**Keywords:** gas absorption, CO_2_ capture, nanofluid, MDEA, membrane wetting, carbon nanotubes (CNTs), global warming

## Abstract

In the present work, membrane resistance was estimated and analyzed, and the results showed that total membrane resistance increased sharply when membrane pores were wetted. For further study, a two-dimensional (2D) mathematical model was developed to predict the chemical absorption of CO_2_ in aqueous methyldiethanolamine (MDEA)-based carbon nanotubes (CNTs) in a hollow fiber membrane (HFM) contactor. The membrane was divided into wet and dry regions, and equations were developed and solved using finite element method in COSMOL. The results revealed that the existence of solid nanoparticles enhanced CO_2_ removal rate. The variables with more significant influence were liquid flow rate and concentration of nanoparticles. Furthermore, there was a good match between experimental and modeling results, with the modeling estimates almost coinciding with experimental data. Solvent enhanced by solid nanoparticles significantly improved the separation performance of the membrane contactor. There was around 20% increase in CO_2_ removal when 0.5 wt% CNT was added to 5 wt% aqueous MDEA.

## 1. Introduction

Gas–liquid hollow fiber membrane (HFM) contactors have attracted the attention of many researchers due to their high interfacial area per unit volume compared to conventional absorption processes. In HFM contactors, mass transfer occurs without dispersion of phases. Shell and tube HFM contactors are employed for the capture of gas impurities, such as CO_2_ and H_2_S, from natural gas and flue gas. The drawbacks of conventional absorption processes, such as the dispersion of phases, can be avoided in HFM contactors. The disadvantages of packed and tray contactor columns, such as emulsion formation, flooding at high gas flow rates, and weeping at low gas flow rates, can also be avoided in HFM contactors [1,2,3,4,5]. The solvents used in CO_2_ absorption and employed by both conventional packed bed columns and membrane contactors are aqueous alkanolamine absorbents. These are the most commonly used absorbents in CO_2_ absorption processes, such as CO_2_ detention from natural gas, flue gas, and biofuels. Despite the advantages of high absorption performance of alkanolamines, they can cause membrane degradation if used in HFM contactors, corrosion problems if used in industrial gas absorbers, and high-energy consumption during solvent regeneration and circulation. Consequently, researchers have continued to look for better absorbents. Ionic liquid is one of the choices proposed and has been used for CO_2_ capture in HFM contactors [6,7,8]. Another alternative is solid nanoparticles dispersed in water (nanofluids), which also makes for an environmentally friendly substitute absorbent. Distilled water has been found to be enhanced by solid nanoparticles, such as carbon nanotubes (CNTs) and silicon oxide (SiO_2_) nanoparticles [9,10,11,12]. In [13], the process of using water-based nanoparticles as solvents was modeled considering diffusion in the radial and axial directions under dry-mode conditions (nonwetting). In [14], CO_2_ was found to be absorbed by water that had been enhanced by nanoparticles, such as aluminum oxide, titanium oxide, and silica, in the concentration range of 0.05–0.2 wt%. In the study, the removal of CO_2_ from a gas mixture of CO_2_/N_2_ using nanofluids (metal oxide in distilled water) in a membrane liquid–gas contacting module was examined. Metal oxide nanoparticles, namely, aluminum oxide (Al_2_O_3_), titanium dioxide (TiO_2_), and silicon dioxide (SiO_2_), in the concentration range of 0.01–0.2 wt% were experimentally investigated. In [15], a numerical model was established to represent the process of capturing CO_2_ from a gas mixture using HMC in distilled water enhanced by carbon nanotubes and nanosilica, mainly at high nanofluid absorbent flow rate. Another study on CO_2_ absorption mechanisms for advancements in the use of nanofluids as absorbent in gas–liquid HFM contactors indicated that absorption of CO_2_ using nanofluids as absorbent was a challenging method for acid gas removal from gas mixtures [16]. CO_2_ absorption from gas mixtures in nanofluids (silica in distilled water) in bubble column absorption has also been investigated [17].

In the area of modeling and simulation of nanofluids, a 2D numerical model was developed for the study of CO_2_ gas capture from a gas mixture in a HFM contactor, with water-based nanofluids used as the liquid absorbent [13,18]. The nanofluids that were composed of 0.05 wt% silica nanoparticles were found to enhance separation by around 15%, while a 30% increase was shown using nanofluids composed of distilled water-based CNTs [18]. In [19], a model describing the absorption of CO_2_ from a gas mixture in a water-based CNT inside a HFM contactor revealed that absorption of CO_2_ was enhanced using solid nanoparticles in water-based solvent. In [20], nanoparticles, such as SiO_2_, Al_2_O_3_, CNT, and Fe_3_O_4_, were dispersed in distilled water at different concentrations (0.02, 0.05, and 0.1 wt%) and in methyldiethanolamine (MDEA) and diethanolamine (DEA) at concentration of 0.02 wt% to form nanofluids. The prepared nanofluids were used in direct contact with pure CO_2_ in a closed vessel at high operating pressures (20, 30, and 40 bar) and a fixed operating temperature of 308 K. In [21], the absorption mechanism of CO_2_ in the presence of nanoparticles compared to fresh water were clarified. In [22], holdups of solid nanoparticles, such as CNT, Fe_3_O_4_, Al_2_O_3_, and SiO_2_, in purified water were used as absorbents for CO_2_ in a pilot HFM contactor. In [23], the effect of nanofluids composed of solid nanoparticles on the mass diffusion rate of CO_2_ absorption were considered. Experiments and modeling of the process performance of CO_2_ capture from gas mixtures using flat sheet membrane contactors were investigated in [24]. In [25], a model of CO_2_ removal from natural gas in HFM contactors was developed and solved using COMSOL software package version 5.4 (Comsol AB, Stockholm, Sweden). The model was comprehensive, taking into account momentum, energy, and mass transport, and the model predictions were within the range of the experimental data. An earlier review looked at the absorption of CO_2_ in membrane contactors [26]. Absorption of CO_2_ from the gas mixture of air/CO_2_ in gas–liquid HFM contactors via nanofluids comprising silica nanoparticles and carbon nanotubes were experimental investigated in [27]. Experimental and theoretical studies have also been performed for the absorption of CO_2_ in a lab-scale reactor using nanofluids composed of carbon nanotubes. Nanosize Al_2_O_3_ particles were used, with water and methanol acting as the base fluids [10,28].

In the present work, a comprehensive 2D mathematical model was developed and solved to study the chemical absorption of CO_2_ from CO_2_/N_2_ gas mixture in aqueous MDEA-based CNT inside a HFM contactor, with the model considering partial wetting. As resistance is mainly located in the liquid phase around solid nanoparticles [18], the membrane module was modeled as five subdivisions: two in the tube side (solid-free region and dense phase), two in the membrane (wetted and dry), and one in the shell side (gas phase). The system governing the material balance equations were numerically solved using the finite element method in COMSOL Multiphysics version 5.4. The model predictions were validated with experimental data available in the literature. CO_2_ concentration profile was investigated in dry and wetted membrane modes. The effect of operating conditions, such as gas flow rate, liquid flow rate, absorbent size, and concentration, on percentage removal of CO_2_ was studied. 

## 2. Mass Transfer Resistance in HFM Contactor

The overall mass transfer resistance ($1/k_{G}$) in a gas–liquid HFM contactor based on the film theory involves three main resistances in series: liquid phase resistance ($1/k_{l}$), membrane resistance ($1/k_{m}$), and resistance of the gas phase boundary layer ($1/k_{g}$). Accordingly, the overall mass transfer resistance in a HFM contactor is expressed as follows:

(1) $$\frac{1}{k_{G}}=\frac{d_{o}}{k_{g}d_{i}}+\frac{d_{o}}{k_{m}d_{lm}}+\frac{1}{mk_{l}}$$

where $k_{g}$ is the mass transfer coefficient in the gas side (m/s); $k_{m}$ is the mass transfer coefficient in the membrane side (m/s); $k_{l}$ is the mass transfer coefficient of the liquid phase; $d_{i}$, $d_{o}$, and $d_{lm}$ are the HFM’s inner, outer, and logarithmic mean diameters, respectively; and m is the dimensionless distribution coefficient at the gas–liquid interface. The mass transfer coefficients $k_{g}$ and $k_{l}$ are based on flow conditions and geometry of the HFM contactor [29].

The mass transfer resistance of membrane ($1/k_{m}$) consists of two resistances: dry membrane resistance ($1/k_{mg}$) and wetted membrane resistance ($1/k_{mw}$), calculated as follows:

(2) $$\frac{1}{k_{mg}}=\frac{\delta \;\tau }{D_{og\;}\varepsilon }$$

(3) $$\frac{1}{k_{mg}}=\frac{\delta \;\tau }{D_{og\;}\varepsilon }$$

(4) $$\frac{1}{k_{m}}=\frac{1-y}{k_{mg}}+\frac{y}{k_{mw}}$$

where y is the fraction length of membrane pores filled with solvents; $k_{mg}$ and $k_{mw}$ are the mass transfer coefficient in gas- and liquid-filled pores, respectively; and ε, δ, and *τ* are the membrane porosity, membrane thickness, and membrane tortuosity, respectively. The liquid mass transfer coefficient is determined using the following relationship:

(5) $$Sh=\sqrt[{3}]{3.57^{3}+1.62^{3}\;Gz}$$

where Sh is the Sherwood number, and Gz is the Gratz number determined as follows:

(6) $$Sh=k_{l}d_{i}/D_{l}$$

And

(7) $$Gz=\frac{V_{l}d_{i}^{2}}{L\;D_{l}}$$

The diffusion coefficient of CO_2_ in the liquid phase is determined by the following relationship [30]:

(8) $$D_{l}\left(m^{2}/s\right)=2.35\times 10^{-6}exp\left(-\frac{2119}{T}\right)$$

The diffusion coefficient of CO_2_ in the dense liquid film around the nanoparticles is expressed as per Equation (9) [31]:

(9) $$D_{n}=D_{l}\left(1+640Re^{1.7}Sc^{1/3}\phi \right)$$

where ϕ is the solid volume fraction, and *Re* is the Reynolds number of the nanosized particles (Brownian motion):

(10) $$Re={\left(\frac{18\;k\;T\rho }{\pi d_{p}\rho_{p}\mu^{2}}\right)}^{0.5}$$

where k is the Boltzmann constant ($1.38\times 10^{-23}\;J/K$), T is the temperature in K, ρ is the liquid density, $d_{p}$ is the particle diameter, $\rho_{p}$ is the particle density, and μ is the viscosity of the liquid.

The Schmidt number $S_{c}$ is

(11) $$Sc=\frac{\mu }{\rho D}$$

The mass transfer coefficient in the gas phase, i.e., the gas stream flowing in the shell side, is determined by [32]:

(12) $$Sh_{s}=0.34Re_{s}^{0.67}Sc_{s}^{0.33}$$

where $Sh_{s}$, $Re_{s}$, and $Sc_{s}$ are the Sherwood number, Reynolds number, and Schmidt number in the shell side, respectively: $Sh_{s}=k_{g}d_{s}/D_{g}$, $Re_{s}=\rho_{g}v_{s}d_{s}/\mu_{g}$, $Sc_{s}=\mu_{g}/\rho_{g}D_{g}$.

The diffusivity in the gas phase ($D_{g}$) is determined using the Chapman–Enskog equation for gas mixture: $D_{g}\;\left(m^{2}/s\right)=1.855\times 10^{-5}$. The density of the CO_2_/N_2_ gas mixture was 343 $\mathrm{kg}/m^{3}$, and the viscosity was $1.65\times 10^{-4}$ Pa·s. The resistances in series were therefore found to be $5.65\times 10^{4}$, $2.67\times 10^{3}$, $5.16\times 10^{6}$, and 165 s/m for the resistances of liquid in the tube side, dry membrane, wetted membrane, and the gas stream in the shell side, respectively. The membrane resistance of the wetted pores was the highest, followed by the liquid phase in the tube side.

## 3. Mathematical Model

**Figure 1:**
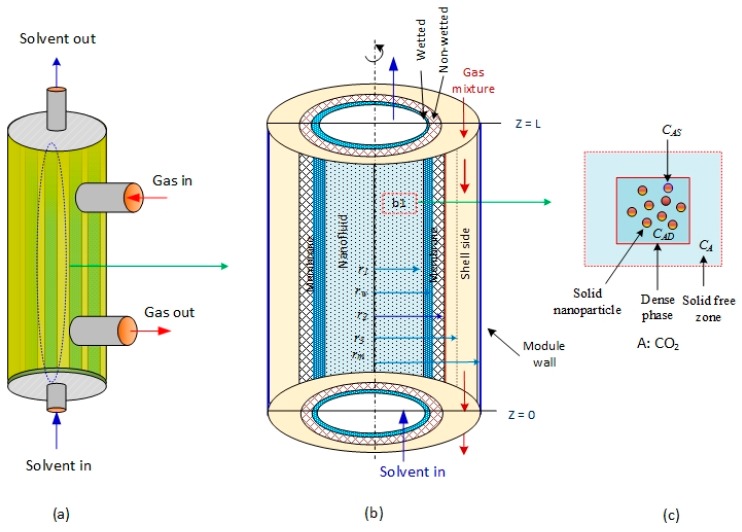
Schematic of the membrane module subdivisions and nanoparticles used in the development of the mathematical model: (**a**) membrane module, (**b**) segment used in modeling, and (**c**) enlargement of box b1 in nanofluid. C_A_, C_As_, and C_AD_ are the CO_2_ concentrations in the solid-free zone, near the solid surface of nanoparticles, and in the dense phase, respectively.

The mathematical model developed in the present work describes the CO_2_ concentration profile in a partially wetted HFM contactor, where CO_2_ is absorbed in aqueous MDEA-based CNT. The membrane contactor comprises three fragments: tube, membrane, and shell section. The tube side, where the absorbent nanofluid passes through, is divided into two subregions: solid-free zone and dense phase. The gas mixture is transported in the shell side counter-currently with the solvent flow direction (Figure 1a). Figure 1b is the subdivision of the membrane module used to develop the mathematical model equations [28]. CO_2_ in the gas phase diffuses through the membrane pores to the nanofluids in the tube side. The ability of N_2_ to dissolve in aqueous MDEA solvents is insignificant relative to CO_2_; subsequently, part of the CO_2_ dissolved in the liquid nanofluid is adsorbed on the surface of the solid nanoparticles, and the other portion reacts with MDEA (Figure 1c). The model considers two main mechanisms that generally take place in the presence of nanofluids: the Brownian motion (random motion of particles suspended in a fluid) and the grazing effect [33,34,35]. The presence of solid nanoparticles in nanofluid enhances the gas absorption due to the adsorption of diffusing gas in the dispersed solid particles. Hence, the gas concentration in the liquid phase near the interface decreases, leading to an increase in the concentration gradient and therefore the absorption rate [16]. Brownian movement increases the velocity near the solid nanoparticle. Microconvection is formed and mass flux dissemination develops, hence altering the diffusion constant [5]. The grazing effect takes place in CO_2_ adsorption at the gas–liquid interface in the presence of solid nanoparticles [36].

The reaction between acid gases (CO_2_) and MDEA has been cited in many articles [3,11,12,37]. There is no hydrogen atom attached to the nitrogen atom in the tertiary amine (MDEA). Hence, CO_2_ first dissolves in the water available with the aqueous alkanolamine to form a bicarbonate ion. It can then react with the amine [38]:

(13) $$CO_{2}+H_{2}O+R_{3}N\to R_{3}NH^{+}+HCO_{3}^{-}$$

The reaction rate of CO_2_ is

(14) $$r_{CO_{2}}=-kC_{CO_{2}}C_{MDEA}$$

Based on Happel’s free surface [39], laminar gas flow surrounds the membrane tubes. At the Happel’s fictional radius ($r=r_{3}$), symmetry is considered. The following assumptions were considered in the model development: steady state operation, constant solvent properties, ideal gas, and nanoparticles as homogeneous. The mathematical equations that describe the system behavior were developed for the tube side (solid nanoparticles and liquid), microporous membrane (wetted and dry), and shell side (flow of gas stream). Accordingly, the model equations for the CO_2_ diffusion path in the tube, membrane, and shell regions are described in the following subsections.

### 3.1. Tube Lumen $\left(0\leq r\leq r_{1}\right)$

The flow of nanofluids (CNT, water, MDEA) in the tube lumen side and the depletion of CO_2_ in the lumen side of the membrane take place by absorption of CO_2_ in water and by the adsorption of CO_2_ on the surface of the nanoparticles and the reaction with aqueous MDEA. Considering the membrane’s partly moisturized section, Equation (15) represents the CO_2_ concentration profile in the solid-free zone ($C_{CO_{2}}$) with dimensionless radius and length:

(15) $$\frac{D_{l}}{R^{2}}\left[\frac{\partial^{2}C_{CO_{2}}}{\partial \varphi^{2}}+\frac{1}{\varphi }\frac{\partial C_{CO_{2}}}{\partial \varphi }\right]+\frac{D_{l}}{L^{2}}\frac{\partial^{2}C_{CO_{2}}}{\partial \psi^{2}}-\frac{u_{z_{t}}}{L}\frac{\partial C_{CO_{2}}}{\partial \psi }=r_{CO_{2}}$$

The concentration of CO_2_ in the dense phase ($C_{CO_{2},D}$) is

(16) $$\frac{D_{n}}{R^{2}}\left[\frac{\partial^{2}C_{CO_{2},D}}{\partial \varphi^{2}}+\frac{1}{\varphi }\frac{\partial C_{CO_{2},D}}{\partial \varphi }\right]+\frac{D_{n}}{L^{2}}\frac{\partial^{2}C_{CO_{2},D}}{\partial \psi^{2}}-\frac{u_{z_{t}}}{L}\frac{\partial C_{CO_{2},D}}{\partial \psi }=R_{d}+r_{CO_{2}}$$

The dimensionless parameters are symbolized by ψ=z/L, $\varphi =r/r_{3}$.

In Equation (15), $D_{l}$ is the diffusion coefficient of the CO_2_ in the solid-free zone in the tube lumen, $D_{n}$ is the diffusion coefficient of CO_2_ in the dense solid phase, $C_{CO_{2},D}$ is the CO_2_ concentration in the dense phase, *L* is the length of the membrane, and *R* is the radius of the hollow fiber. The adsorption rate, $R_{d}$, is as follows:

(17) $$R_{d}=k_{p}a_{p}\left(C_{CO_{2},D}-C_{CO_{2},S}\right)$$

where $k_{p}$ is the solid–liquid mass transfer coefficient (m/s), $a_{p}$ is the solid–liquid interfacial area ($m^{2}/m^{3})$, $C_{CO_{2},D}$ is the solute concentration in the suspension ($\mathrm{mol}/m^{3}$), $C_{CO_{2},S}$ is the solute concentration at the interface of the particles ($\mathrm{mol}/m^{3}$).

The adsorbed amount of CO_2_ on the solid nanoparticles per unit mass of particles, $q\;\left(\frac{\mathrm{mol}}{\mathrm{kg}}\right),$ can be given by

(18) $$\phi \rho_{p}\frac{v_{zt}}{L}\frac{\partial q}{\partial \varphi }=k_{p}a_{p}\left(C_{CO_{2},D}-C_{CO_{2},S}\right)$$

where $v_{zt}$ is the velocity in the tube side. The $k_{p}$ value is estimated from Equation (19). The following correlation is used for mass transfer for flow past single spheres [32]:

(19) $$Sh=2+0.552Re^{0.5}Sc^{0.33}$$

The value of Sh was found to be 2.08, hence

(20) $$Sh=\frac{k_{p}d_{p}}{D_{CO}{}_{2}}=2.08$$

Then, the $k_{p}$ value is determined as follows:

(21) $$k_{p}=2.08\times D_{CO_{2}}/d_{p}$$

where $D_{CO_{2}}$ is the CO_2_ diffusivity in the solid-free zone. The adsorption of the CO_2_ onto the surface of nanoparticles (q) is described by the Langmuir isotherm equation as follows:

(22) $$q=q_{m}\frac{k_{d}\;C_{CO_{2},s}}{1+k_{d}\;C_{CO_{2},s}}$$

where $q\;\left(\frac{\mathrm{mol}}{\mathrm{kg}}\right)$ is the adsorbed amount of CO_2_ on solid surface per unit mass of particle, $q_{m}$ is the highest quantity of adsorbed gas solute, and $k_{d}\;\left(m^{3}/\mathrm{mol}\right)$ is the Langmuir coefficient. The velocity distribution inside the tube ($v_{zt}$) is assumed to follow Newtonian laminar flow as per Equation (23).

(23) $$v_{zt}=2v_{zt,avg}\left(1-{\left(\frac{r}{r_{1}}\right)}^{2}\right)$$

The appropriate boundary conditions are as follows:

(24) $$\mathrm{inlet}\;\mathrm{of}\;\mathrm{liquid},\;z=0,\;C_{CO_{2}}=C_{CO_{2},D}=0\;(\mathrm{fresh}\;\mathrm{solvent})$$

(25) $$\mathrm{exit}\;\mathrm{of}\;\mathrm{liquid},\;z=L,\;\frac{\partial C_{CO_{2}}}{\partial \psi }=\frac{\partial C_{CO_{2},D}}{\partial \psi }=0\;(\mathrm{convective}\;\mathrm{flux})$$

(26) $$\mathrm{tube}\;\mathrm{center},\;r=0,\;\frac{\partial C_{CO_{2}}}{\partial \varphi }=\frac{\partial C_{CO_{2}D}}{\partial \varphi }=0\;(\mathrm{axial}\;\mathrm{symmetry})$$

(27) $$\mathrm{inner}\;\mathrm{radius}:\;r=r_{1},\;C_{CO_{2}}=C_{CO_{2},wm}\;(\mathrm{wetted}\;\mathrm{membrane})$$

### 3.2. Membrane $\left(r_{1}\leq r\leq r_{2}\right)$

#### 3.2.1. Wetted Membrane Section ($r_{1}\leq r\leq r_{w})$

The steady-state material balance for the transport of CO_2_ is inside the wetted portion of the membrane (there is reaction but no convective term), and diffusion takes place in the wetted membrane. The CO_2_ transport in the wetted membrane portion is described by Equation (28):

(28) $$\frac{D_{CO_{2,}wm}}{R^{2}}\left[\frac{\partial^{2}C_{CO_{2},wm}}{\partial \varphi^{2}}+\frac{1}{\varphi }\frac{\partial C_{CO_{2},wm}}{\partial \varphi }\right]+\frac{D_{CO_{2},wm}}{L^{2}}\frac{\partial^{2}C_{CO_{2},wm}}{\partial \psi }=\epsilon R_{CO_{2}}$$

where $C_{CO_{2},wm}$ is the concentration of CO_2_ in the wetted portion of the membrane segment. The diffusivity of CO_2_ in the wetted membrane section is determined as follows: $D_{CO_{2},wm}=D_{l\;}\varepsilon /\tau$.

The appropriate boundary conditions in wetted membrane (wm) zone are as follows:

(29) $$\mathrm{tube}\text{–}\mathrm{wetted}\;\mathrm{membrane}\;\mathrm{interface},\;r=r_{1},\;C_{CO_{2},wm}=C_{CO_{2}}$$

(30) $$\mathrm{wetted}\text{–}\mathrm{dry}\;\mathrm{membrane}\;\mathrm{interface},\;r=r_{2},\;C_{CO_{2},wm}=m\;C_{CO_{2},m}$$

(31) $$\mathrm{hollow}\;\mathrm{fiber}\;\mathrm{membrane}\;\mathrm{at}\;z\;=\;0,\;\frac{\partial C_{CO_{2},wm}}{\partial \psi }=0$$

(32) $$\mathrm{hollow}\;\mathrm{fiber}\;\mathrm{membrane}\;\mathrm{at}\;z\;=\;L,\;\frac{\partial C_{CO_{2},wm}}{\partial \psi }=0$$

#### 3.2.2. Dry Section of the Membrane ($r_{w}\leq r\leq r_{2})$

The CO_2_ concentration in the dry part of the membrane, where there is no reaction and no convective term and only diffusion takes place, is calculated as follows:

(33) $$\frac{D_{m}}{R^{2}}\left[\frac{\partial^{2}C_{CO_{2},m}}{\partial \varphi^{2}}+\frac{1}{\varphi }\frac{\partial C_{CO_{2},m}}{\partial \varphi }\right]+\frac{D_{m}}{L^{2}}\frac{\partial^{2}C_{CO_{2},m}}{\partial \psi }=0$$

The arbitrary boundary conditions are as follows:

(34) $$\mathrm{wetted}\text{–}\mathrm{dry}\;\mathrm{membrane}\;\mathrm{interface},\;r=r_{w},\;C_{CO_{2},m}=C_{CO_{2},wm}/m$$

(35) $$\mathrm{membrane}\text{–}\mathrm{shell}\;\mathrm{interface},\;r=r_{2},\;C_{CO_{2},m}=C_{CO_{2},g}$$

(36) $$\mathrm{membrane}\;\mathrm{bottom}\;\mathrm{edge},\;z=0,\;\frac{\partial C_{CO_{2},m}}{\partial \psi }=0$$

(37) $$\mathrm{membrane}\;\mathrm{top}\;\mathrm{edge},\;z=L,\;\frac{\partial C_{CO_{2},m}}{\partial \psi }=0$$

The diffusivity of CO_2_ in the nonwetted membrane section is $D_{m}=D_{g}\varepsilon /\tau$, where ε and τ are the porosity and tortuosity of the membrane, respectively. 

### 3.3. Shell Side $\left(r_{2}\leq r\leq r_{3}\right)$

The material balance of the CO_2_ in the shell side ($C_{CO_{2},g}$), bounded between the membrane’s outer skin layer and Happel’s free surface model at steady state, is as follows:

(38) $$\frac{D_{g}}{R^{2}}\left[\frac{\partial^{2}C_{CO_{2},g}}{\partial \varphi^{2}}+\frac{1}{\varphi }\frac{\partial C_{CO_{2},g}}{\partial \varphi }\right]+\frac{D_{g}}{L^{2}}\frac{\partial^{2}C_{CO_{2},g}}{\partial \psi^{2}}-\frac{v_{zs}}{L}\frac{\partial C_{CO_{2},g}}{\partial \psi }=0$$

The arbitrary boundary conditions are

(39) $$\mathrm{gas}\;\mathrm{inlet}\;\mathrm{side},\;z=L,\;C_{CO_{2},g}=C_{CO_{2},0}\;(\mathrm{inlet}\;{\mathrm{CO}}_{2}\;\mathrm{concentration})$$

(40) $$\mathrm{gas}\;\mathrm{exit}\;\mathrm{side},\;z=0,\;\frac{\partial C_{CO_{2},g}}{\partial \psi }=0\;(\mathrm{convective}\;\mathrm{flux})$$

(41) $$\mathrm{free}\;\mathrm{surface},\;r=r_{3},\;\frac{\partial C_{CO_{2},g}}{\partial \psi }=0\;(\mathrm{symmetry})$$

(42) $$\mathrm{shell}\text{–}\mathrm{membrane}\;\mathrm{interface},\;r=r_{2}\;C_{CO_{2},g}=C_{CO_{2},m}$$

The axial velocity in the shell side is expressed by Happel’s free surface model [39]:

(43) $$v_{zs}=2V_{zs,avg}\left[1-{\left(\frac{r_{2}}{r_{3}}\right)}^{2}\right]\frac{\left({\left(\frac{r}{r_{3}}\right)}^{2}-{\left(\frac{r_{2}}{r_{3}}\right)}^{2}+2\ln\left(\frac{r_{2}}{r}\right)\right)}{\left(3+{\left(\frac{r_{2}}{r_{3}}\right)}^{4}-4{\left(\frac{r_{2}}{r_{3}}\right)}^{2}+4\ln\left(\frac{r_{2}}{r_{3}}\right)\right)}$$

Table 1 lists the parameters used in the numerical solution of the model equations. COSMOL Multiphysics 5.4 was employed to solve the set of partial differential equations.

The properties of the solid CNT are listed in Table 2. 

## 4. Results and Discussion

Analysis of the mass transfer resistance in the HFM contactor (liquid, membrane, and gas phases) revealed that wetting of membrane pores by the absorbent liquid led to a high increase in the total membrane resistance and hence decreased the percentage removal of CO_2_. This can be attributed to the presence of stationary liquid in the wetted piece of the membrane pores, which consequently caused a delay in the CO_2_ transport in the membrane holes. Accordingly, the concentration of the CO_2_ at the membrane–liquid interface decreased, and the CO_2_ removal efficiency also decreased [44]. In the absorbent liquid nanofluid stream in the tube lumen, the liquid–solid mass transfer resistance was low due to the nanosize of the solid nanoparticle; hence, the mass transfer resistance was concentrated in the solid-free liquid region [22]. 

**Figure 2:**
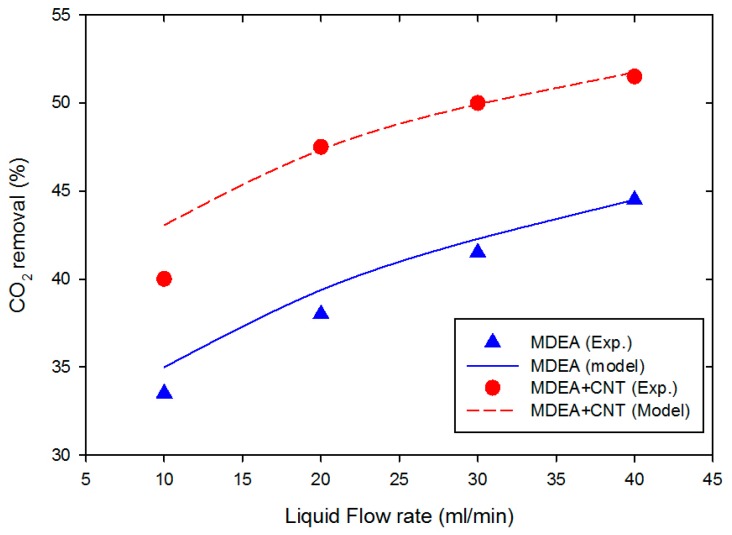
Comparison of developed model predictions (present model) with experimental data [40] for aqueous methyldiethanolamine (MDEA) solution (5 wt%, 0% carbon nanotubes (CNT), with the balance being water) and after adding CNT (5 wt% MDEA, 0.5 wt% CNT, with the balance being water). Gas and liquid flow rates, 10 ml/min; wetting, 0.2%; atmospheric pressure and lab temperature, 25 °C.

In order to check the accuracy of the developed mathematical model, the model was validated with our published experimental data obtained for CO_2_ absorption from a gas mixture consisting of CO_2_/N_2_ in aqueous alkanolamine solution with and without solid nanoparticles of carbon nanotubes dispersed in the aqueous MDEA [40]. The model predictions (solid line) for both amine-free aqueous solution (5 wt% MDEA, with the balance being water) and aqueous amine solution with carbon nanotubes (5 wt% MDEA, 0.5 wt% CNT, with the balance being water) are depicted in Figure 2. There was an excellent match between the experimental and simulation results, confirming the promising predictions of the developed model. The deviation of the predicted results from the experimental data was measured using the root mean square error (RMSE) as follows:

(44) $$\mathrm{RMSE}={\left(\frac{\sum E_{i}^{2}}{n}\right)}^{0.5}$$

where n is the sum of the investigated data points, and $E_{i}^{2}$ is the square of the error between predicted results from the model and experimental data point. The relative error ($E_{i})$ was measured as per Equation (45):

(45) $$E_{i}=\frac{y_{exp}-y_{mod}}{y_{exp}}$$

where $y_{exp}$ and $y_{mod}$ are the experimental and model prediction data points, respectively. The value of the RMSE for the system of aqueous 5 wt% MDEA/water was around 0.01, while it was 0.007 for CNT/MDEA/water. Results revealed that the CO_2_ removal rate increased when liquid flow rate increased. This can be attributed to the fact that the thickness of the liquid boundary layer decreased with the increase in liquid flow rate, and the decrease in the liquid boundary layer in the hollow fiber increased the CO_2_ diffusion rate into the absorbents. Consequently, the liquid–gas border was kept at low CO_2_ concentration (high concentration gradient), which improved the percentage removal of CO_2_.

**Figure 3:**
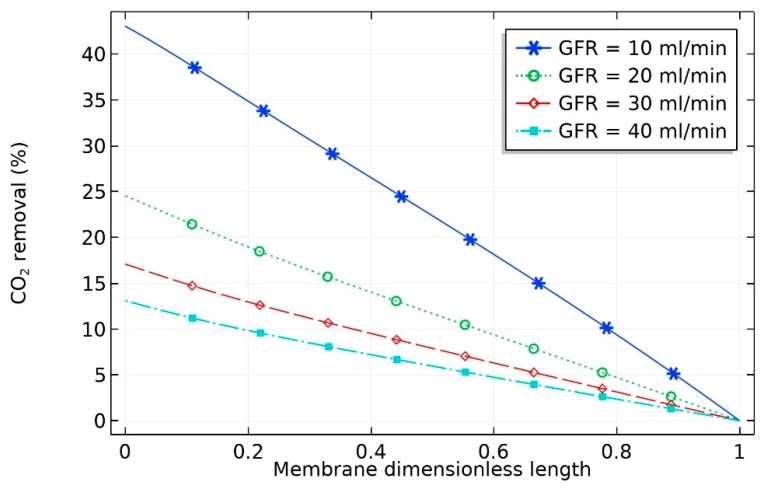
Effect of inlet gas volumetric flow rate (GFR) on the percentage removal of CO_2_ at a fixed liquid flow rate (10 mL/min) and solvent composition (0.5 wt% CNT, 5 wt% MDEA, and 20 vol% CO_2_).

At constant liquid flow rate, the effect of flow rate of gas (GFR) on the percentage removal of CO_2_ is depicted in Figure 3. It can be seen that the percentage removal at a fixed liquid flow rate was not evenly spaced; rather, as expected, the percentage removal of carbon dioxide decreased at high gas flow rates. With the increase in gas volumetric flow rate from 10 to 20 mL/min, a sharp decrease in percentage removal of CO_2_ from 45 to 25% occurred. This can be attributed to the decrease in the residence time of the gas stream in the shell side of the hollow fiber membrane, which negatively influenced the effectiveness of CO_2_ separation in the membrane contactor. The percentage removal of CO_2_ (η) can be obtained as per Equation (46):

(46) $$\eta =\frac{C_{in}Q_{in}-C_{out}Q_{out}}{C_{in}Q_{in}}\times 100$$

**Figure 4:**
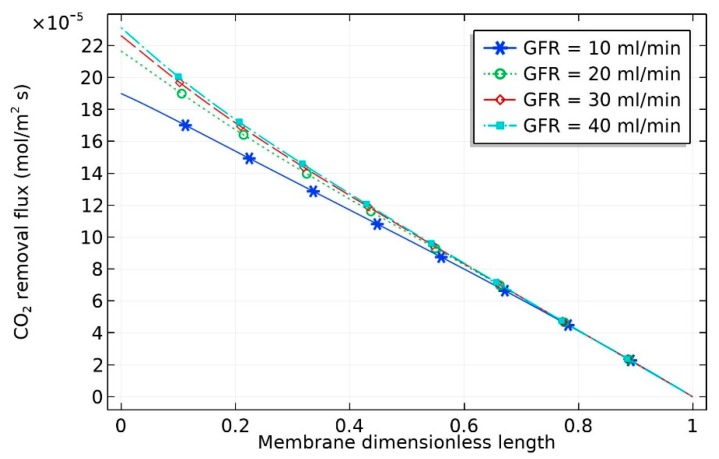
Effect of inlet GFR on the percentage removal of CO_2_ in the hollow fiber membrane (HFM) contactor system (0.5 wt% CNT, 5 wt% MDEA, 20 vol% CO_2_).

The influence of feed flow rate of gas on CO_2_ removal flux is shown in Figure 4. Increasing gas velocity improved the gas mass transfer coefficient and hence increased the CO_2_ withdrawal flux [24]. This can be attributed to the fact that the gas mass transfer coefficient is directly related to the gas velocity [18]. The CO_2_ removal flux, which is used to indicate the process efficiency, can be estimated by the following equation:

(47) $$J_{CO_{2}}\left(\frac{\mathrm{mol}}{m^{2}\cdot s}\right)=\frac{\left(y_{CO_{2},in}Q_{in}-y_{CO_{2},out}\;Q_{out}\right)\times 273.15\times 1000}{22.4\times T_{g}\times A_{T}}$$

where $J_{CO_{2}}$ is the CO_2_ removal flux; $y_{CO_{2},in}$ and $y_{CO_{2},out}$ are the inlet and exit CO_2_ mole fraction, respectively; $Q_{in}$ and $Q_{out}$ (m^3^/s) represent the inlet and exit volumetric flow rate of gas in the gas phase, respectively; $T_{g}\;\left(K\right)$ is the real gas temperature; and $A_{T}\;\left(m^{2}\right)$ represents the membrane area at the liquid–gas interface.

**Figure 5:**
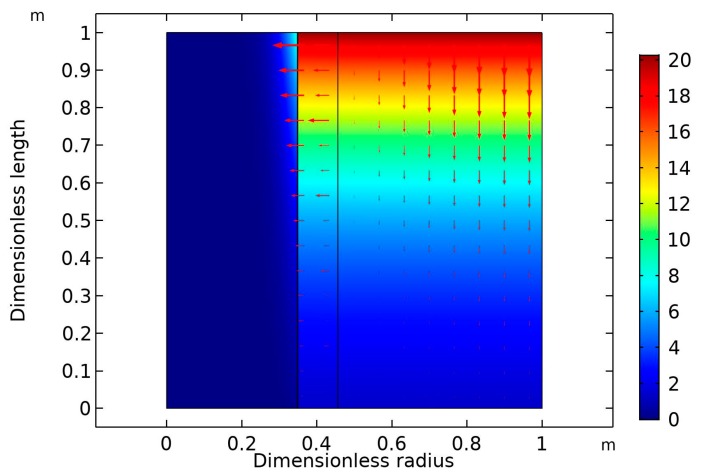
Surface plot diagram of the CO_2_ volume percentage through the HMC at equal inlet gas and liquid flow rates (10 mL/min), nanofluid composition (0.5 wt% CNT, 5 wt% MDEA, with the balance being water), and feed gas composition (20 vol% CO_2_). Arrows represent convective flux.

As can be seen from the figure, there was a significant increase in CO_2_ removal flux when the liquid flow rate increased from 10 to 20 mol/min, with the removal flux increasing from $1.9\times 10^{-4}$ to $2.2\times 10^{-4}$ mol/m^2^·s. By contrast, the increase in molar flux was insignificant when the liquid flow rate increased from 20 to 40 mL/min. This can be attributed to the drop in the CO_2_ concentration gradient with increasing liquid flow rate. The crosswise contour of CO_2_ concentration in the HFM contactor is predicted in Figure 5. The inlet liquid and gas flow rates were both fixed at 10 ml/min. The feed stream contained 20 vol% CO_2_ in the CO_2_/N_2_ gas mixture. The inlet nanofluid contained dispersed CNT in aqueous MDEA solution (5 wt% MDEA, 0.5 wt% CNT, with the balance being water). The diagram reveals that there was a drop in the CO_2_ volume in the inlet gas stream. It dropped downward in the shell side of the membrane from 20 to around 5 vol%.

**Figure 6:**
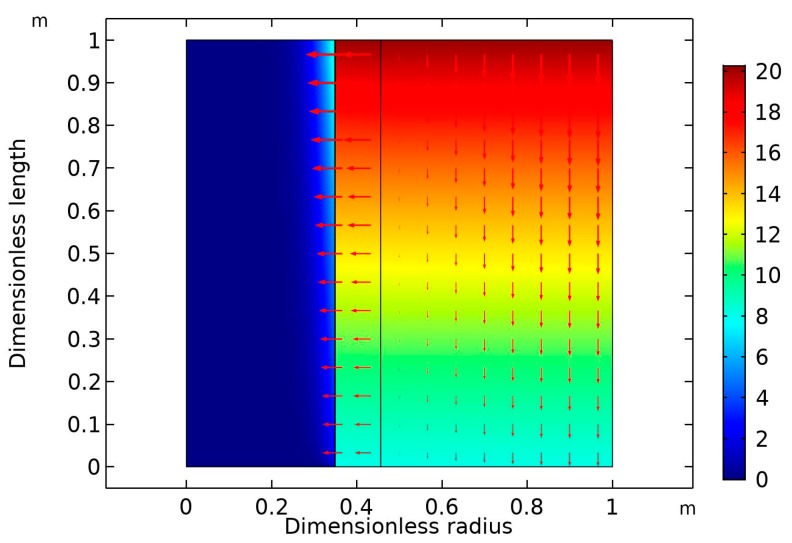
Surface plot of the CO_2_ volume percentage through the membrane separation process at constant gas and liquid flow rates (30 and 10 mL/min, respectively), nanofluid composition (0.5 wt% CNT), and liquid and gas feed composition (5 wt% MDEA, 20 vol% CO_2_). Arrows represent convective flux.

Figure 6 is the surface plot of the CO_2_ volume at high gas flow rate (30 mL/min) and fixed liquid flow rate (10 mL/min). As expected, the higher the gas velocity, the lower was the CO_2_ removal rate [36]. In this case, the percentage removal of CO_2_ dropped from 90% (GFR = 10 mL/min) to around 60% (GFR = 30 mol/min).

**Figure 7:**
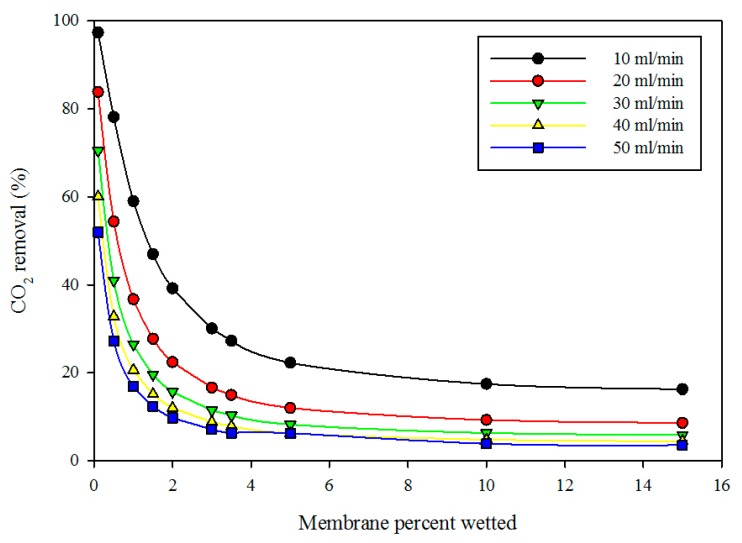
Model predictions of the effect of wetted membrane percentage on the percentage removal of CO_2_ at variable gas flow rate and fixed liquid flow rate (10 mL/min).

Figure 7 displays the effect of membrane wetting on the percentage removal of CO_2_ at variable gas volumetric flow rates and fixed liquid flow rate (10 mL/min). The diagram reveals that the percentage wetting of membrane had a significant impact on the percentage removal of CO_2_. As the wetted membrane portion increased, the percentage removal of CO_2_ decreased. This can be attributed to the fact that the membrane resistance increased with membrane wetting because the diffusion coefficient of gas in the wetted membrane (liquid-filled pores) was much lower than the CO_2_ diffusion in the dry membrane (gas-filled pores). At fixed membrane wetting, as the gas flow rate increased, the percentage removal of CO_2_ decreased. This can be attributed to the fact that, as the gas flow rate increased, the gas residence time in the membrane shell side decreased, thereby reducing the chance of gas molecules to come into direct contact with liquid at the gas-liquid interface.

**Figure 8:**
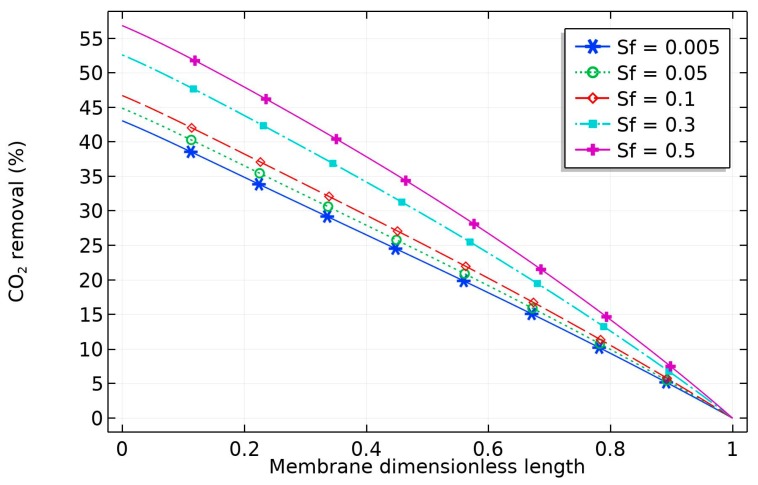
Effect of CNT volume fraction on the percentage removal of CO_2_. Liquid and gas flow rates were both 10 mL/min. The solvent contained variable volume fraction of CNT, 5 wt% MDEA, with the balance being water. The feed gas contained 20 vol% CO_2_, with the balance being N_2_.

The effect of nanoparticle volume fraction on the solvent is depicted in Figure 8. The diagram reveals that the percentage removal of CO_2_ increased with solid nanoparticles, which can be attributed to the grazing effect (increase in the amount of CO_2_ adsorbed into the surface of the CNT). The increase would be limited by obtaining a homogeneous solvent, but this is not achievable for high CNT concentration [36].

## 5. Conclusions

The present work aimed to study the chemical absorption process of CO_2_ from CO_2_/N_2_ gas mixture in aqueous MDEA-based CNT in a HFM contactor. The membrane resistance was studied, and the results showed that wetted membrane pores significantly increased the membrane resistance. A steady-state 2D mathematical model was developed considering partially wetted membrane. The effect of gas flow rate, liquid flow rate, nanoparticle volume fraction, and membrane wetting on CO_2_ removal rate and CO_2_ removal flux was investigated. The tube side was modeled as a solid-free zone and a dense phase, while the shell side was modeled as a single gas phase. The model governing the equations were solved using finite element method built in COSMOL Multiphysics version 5.4. The predicted results revealed that the existence of solid nanoparticles enhanced the CO_2_ removal rate. The present model offered a good basis to explore the performance of CO_2_ capture in the presence of nanoparticles in reactive solvent (MDEA). The CO_2_ removal rate increased with solid concentration. The simulation results revealed that liquid flow rate and concentration of nanoparticles had a strong impact on the CO_2_ absorption and hence the CO_2_ removal efficiency and removal flux. The model predictions and the experimental data were compared and found to be in good agreement. 

## Figures and Tables

**Table 1:** Characteristics of polyvinylidene fluoride (PVDF) membrane module and operating parameters.

Parameter	Value	Reference
Fiber inner radius (m)	$2.1\times 10^{-4}$	[40]
Fiber outer radius (m)	$5.5\times 10^{-4}$	[40]
Module diameter (m)	0.08	[40]
Module length (m)	0.21	[40]
Total number of fibers	11	[40]
$D_{l}\;\left(m^{2}/s\right)$	$2.35\times 10^{-6}e^{\frac{-2199}{T}}$	[41]
$D_{CO_{2},g\;}\left(m^{2}/s\right)$	$1.855\times 10^{-5}$	[42]
$D_{CO_{2},m}\;\left(m^{2}/s\right)$	$D_{CO_{2},g}\varepsilon /\tau$	Estimated
$D_{CO_{2},wm}\;\left(m^{2}/s\right)$	$0.5D_{l}$	Estimated
m=1/H	$H=2.82\times 10^{6}\exp\left(\frac{-2044}{T}\right)/RT$	[41]
Porosity, *ε*	0.46	[40]
Tortuosity, *τ*	(2−ε)/ϵ	[24]
$k\;(m^{3}/\left(\mathrm{kmol}\cdot s\right)$	$8.741\times 10^{12}\exp\left(-\frac{8625}{T}\right)$	[43]

**Table 2:** Properties of the solid carbon nanotubes (CNT).

Morphology	Tubular	References
Particle density, $\rho_{p}$	2200 kg/m^3^	[40]
Particle diameter, dp	8 nm	[40]
Liquid-solid mass transfer coefficient, $k_{p}$	2.6 × 10^−3^ m/s	[28]
Maximum adsorbed, $q_{m}$	29.45 mol/kg	[18]
Specific surface area, $a_{p}$	$S_{p\;\times \;}\rho_{p}$ [1/m]	Estimated
Isotherm constant, Langmuir, $k_{d}$	0.49 m^3^/kmol	[18]
CNT weight percent, wt%	0.5%	[40]
Solid surface area, $S_{p}$	500 m^2^/g	[18]
